# Advantages of carotid revascularization in the elderly

**DOI:** 10.1186/1471-2482-13-S1-A47

**Published:** 2013-09-16

**Authors:** Luciana Tromba, Sara Blasi, Gregorio Patrizi, Dimitra Kiltzanidi, Jamila Ben Hamida, Francesca Frezzotti, Giorgio Di Rocco, Adriano Redler

**Affiliations:** 1 Dipartimento di Scienze Chirurgiche , Divisione di Chirurgia Generale G , Policlinico Umberto I di Roma, Viale Regina Elena 324, 00161, Italy

## Introduction

Atherosclerosis is an aging disease and mortality and morbidity related to cerebral ischemia concern mainly elderly. The Framingham Study showed that: at 50 years <1% of the population presents a stenosis ≥ 50%, at 75 the percentage rises to 7% and at 80 it reaches 10% 1. 8% of deaths and 74% of all admissions related to this pathology concern the over sixty-five population. In the last ten years, carotid revascularization globally increased by 48% and the age group most represented is the VII decade. Moreover, data show a growing trend for revascularization in asymptomatic patients. Carotid stenosis are not only responsible of the embolic risk, as it was always thought, but also of the changes of intracranial blood flow and self-regulation (the ability to preserve a constant cerebral perfusion through the function of the microcirculatory unit that affects flow resistance by bio-metabolic and neuro-endocrine mechanisms and reacts to stimulatory inputs that can be pressure, chemical, psychological stimuli) 2. If such ability is lost, parenchyma becomes vulnerable to ischemic or pressure damage. Patients with asymptomatic carotid stenosis and impaired self-regulation present a reduced cognitive function compared to subjects of the same age and without stenosis and a proper self-regulation. Several authors demonstrated an improved cognitive function following carotid revascularization in subjects presenting preoperative cerebral decay. Aim of this retrospective study is to: 1)- identify the criteria that determined the therapeutic choice in the elderly to avoid the embolic risk; 2)- verify the intracranial hemodynamic improvements following revascularization to reduce cognitive loss.

## Methods

From 2009 to 2012 in our division of General Surgery (Department of Surgical Sciences, Policlinico Umberto I, Roma), globally 52 patients (35 men, age 69-82 years and 17 women, age 71-86 years) underwent carotid revascularization by endarterectomy (CEA) or stenting. Symptomaticity of the plaque was assessed in all patients, then routine blood tests, chest film, EKG and echocardiogram were performed. All patients underwent also carotid echo-color-Doppler to identify the plaque and to define its localization, morphology, structure, echogenicity, surface, percentage of stenosis and possible complications such as ulcers and intraplaque hemorrhage. All patients underwent cerebral CT scan. Furthermore, all patients underwent transcranic color-Doppler to evaluate possible stenosis of the intracranial arteries and to assess compensation and self-regulation mechanisms.

## Results

30 of the 52 subjects were asymptomatic, 18 presented previous TIA, 2 referred a RIND in the previous six months and 2 a TIA one year before. Cerebral CT scan showed ischemic lesions in all cases and in 2 clinically asymptomatic patients such lesions were extensive. In asymptomatic patients the plaques were hypoechogenous in 50% of cases and in the remaining 50%, they were hyperechogenous with an irregular surface, presenting intraplaque hemorrhage in 2 cases, with stenosis ranging from 60 to 85%. Intracranial evaluation showed in about 20% of cases preoperative insufficient compensation that improved after revascularization. Moreover, in all cases middle cerebral artery flow increased and cerebral blood flow reserve improved. Such findings were more evident, more than normally, in younger and lasted longer, even for months. In the elderly, the postoperative improvement decreased faster to stabilize by the way at a level higher than the preoperative one.

## Conclusions

Severe stenoses, high-risk plaques or carotid obstructions can remain asymptomatic until the neurological event, not always reversible, frequently disabling or even fatal. Therefore prevention and early diagnosis especially in the asymptomatic period is mandatory. The risk arising from a carotid stenosis is not only related to its morphological aspects, its structure and percentage of stenosis but also to the efficiency of the cerebral supplying circuits and to cerebral reserve flow which is a factor independent from the risk of stroke, both in symptomatic and asymptomatic patients [3]. The definition of asymptomaticity is not only clinical. A clinical asymptomatic stenosis can be considered symptomatic if ipsilateral ischemic lesions exist. Several studies assessed that removing a plaque means reducing the embolic risk and also hemodynamic deterioration [4]. The increase of revascularization, especially in the elderly, is then related to an increased interest in prevention, improvement and diffusion of diagnostic tools, reduction of the perioperative risk and increased life expectancy. Cognitive function in a 4 years follow-up after EAC show cerebral decay in symptomatic stenosis but not in the asymptomatic ones. We therefore believe that carotid revascularization before the onset of symptoms can help preventing cerebral decay.
